# Delivering informatics for clinical research in developing countries

**DOI:** 10.1186/1471-2105-13-S12-A24

**Published:** 2012-07-31

**Authors:** Jonathan Babbage, Naga S Nagisetty, Steven P Larmore, Jacqueline Fiore

**Affiliations:** 1 Clinical and Translational Sciences Institute, Michigan State University, East Lansing, MI 48824, USA; 2 Malaria Alert Centre, University of Malawi College of Medicine, Blantyre, Malawi

## Background

Not all diseases exist in locations where they are easy to study. The International Centers of Excellence for Malaria Research [1] bring the critical infrastructure necessary for research to parts of these endemic regions. We have implemented some of the existing health care informatics capabilities that deliver on the specific research requirements in a fast, accurate, and secure way in a developing country [2]. Our approach has been modified to work around some of the unique conditions present in these areas. Challenges have included identifying appropriate hardware and software for the studies, delivering the hardware to Malawi (Southern Africa), limited network connectivity, mobility constraints, GPS data collection, barcode generation, local customs, and limited availability of information technology staff.

## Materials and methods

We present an informatics approach that was implemented in Malawi to accommodate six different facilities, over twenty staff members doing data entry, and one field data collector that travels to subjects' houses. The approach we implemented included a server installation at the Malaria Alert Center (MAC) in Blantyre, offline data capture devices (running a customized version of REDCap [3]), sample barcoding, and integrated location capture for the geographical statistics. The server acts as the central repository for both the study metadata and the data from the offline data capture systems. The data collection team will use the offline device to collect the data while at a regional health facility or district hospital. They return to the MAC at the end of the day and migrate the data to the server. The customized version of REDCap acts as the offline EDC and GPS data collection tool which allows for subject identification using a barcode as opposed to hand entered ids. We provided necessary training sessions to the study personnel on configuring the data capture systems for individual studies, managing the infrastructure, and using the different components to capture the data effectively. Two months after launch we are approaching 10,000 subjects enrolled and their corresponding samples. We will discuss successes and setbacks we have experienced.

**Figure 1:**
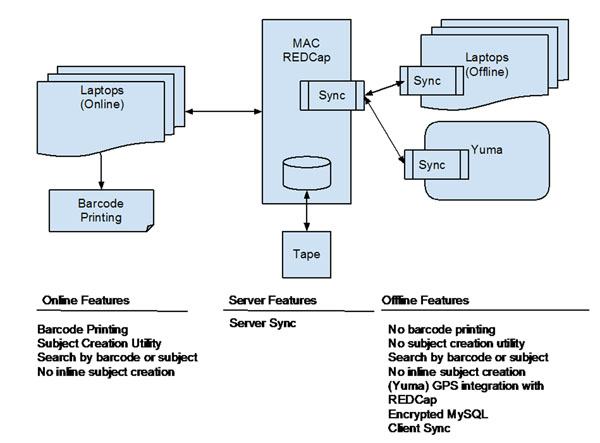
Infrastructure diagram.
